# Supplementary figures and images for: Filarial DAF-12 sense the host serum to resume iL3 development during infection
Source: PLoS Pathog. 2023 Jun 20;19(6):e1011462. doi: 10.1371/journal.ppat.1011462 (PMC10313052; doi:10.1371/journal.ppat.1011462)

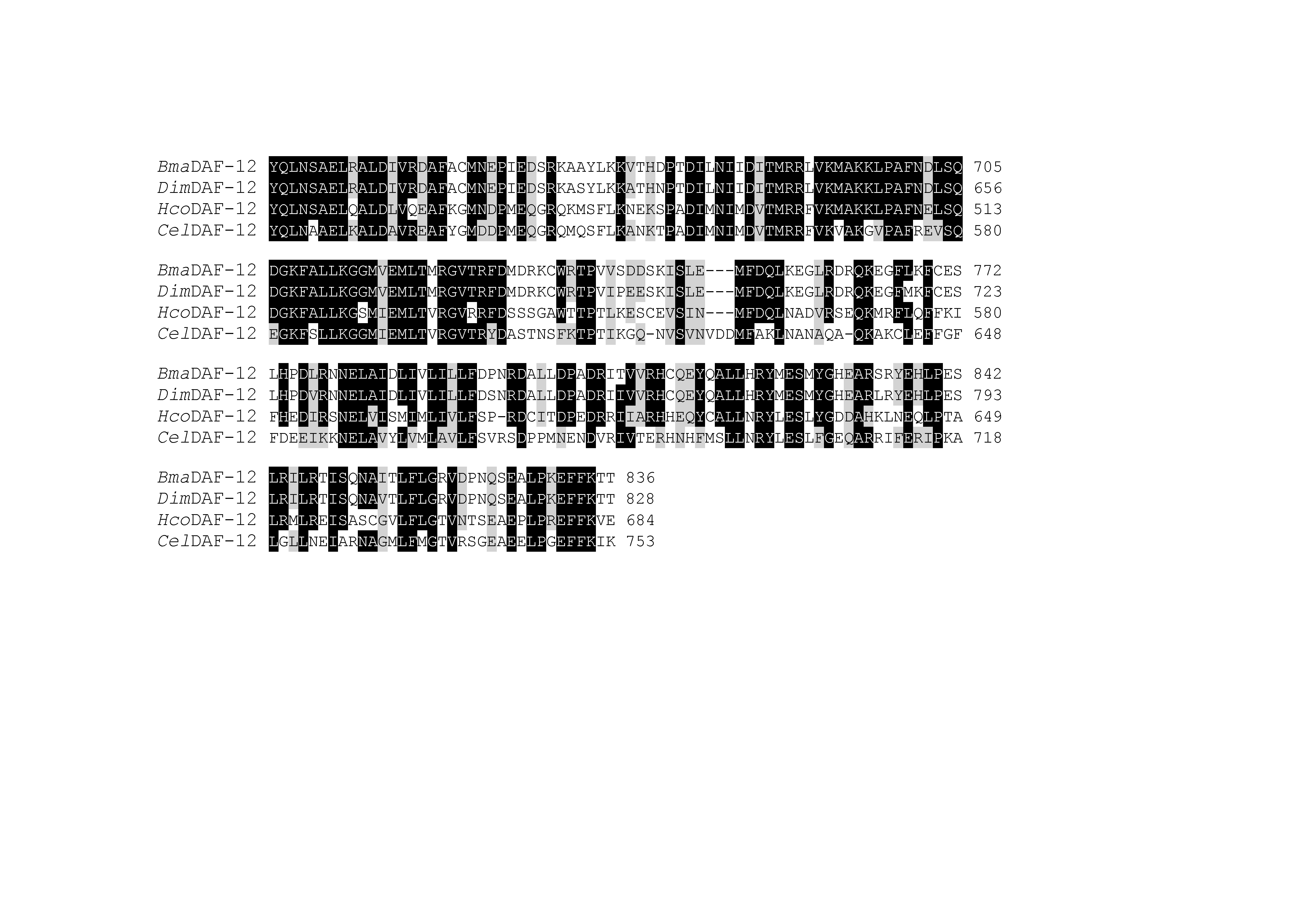

Supplement: S1 Fig — Multiple sequence alignment was performed with the MAFFT program from MyHits. Identical (black) and similar (grey) residues have been colored coded with the Color Align Conservation program. (TIFF) [file ppat.1011462.s001.tiff]

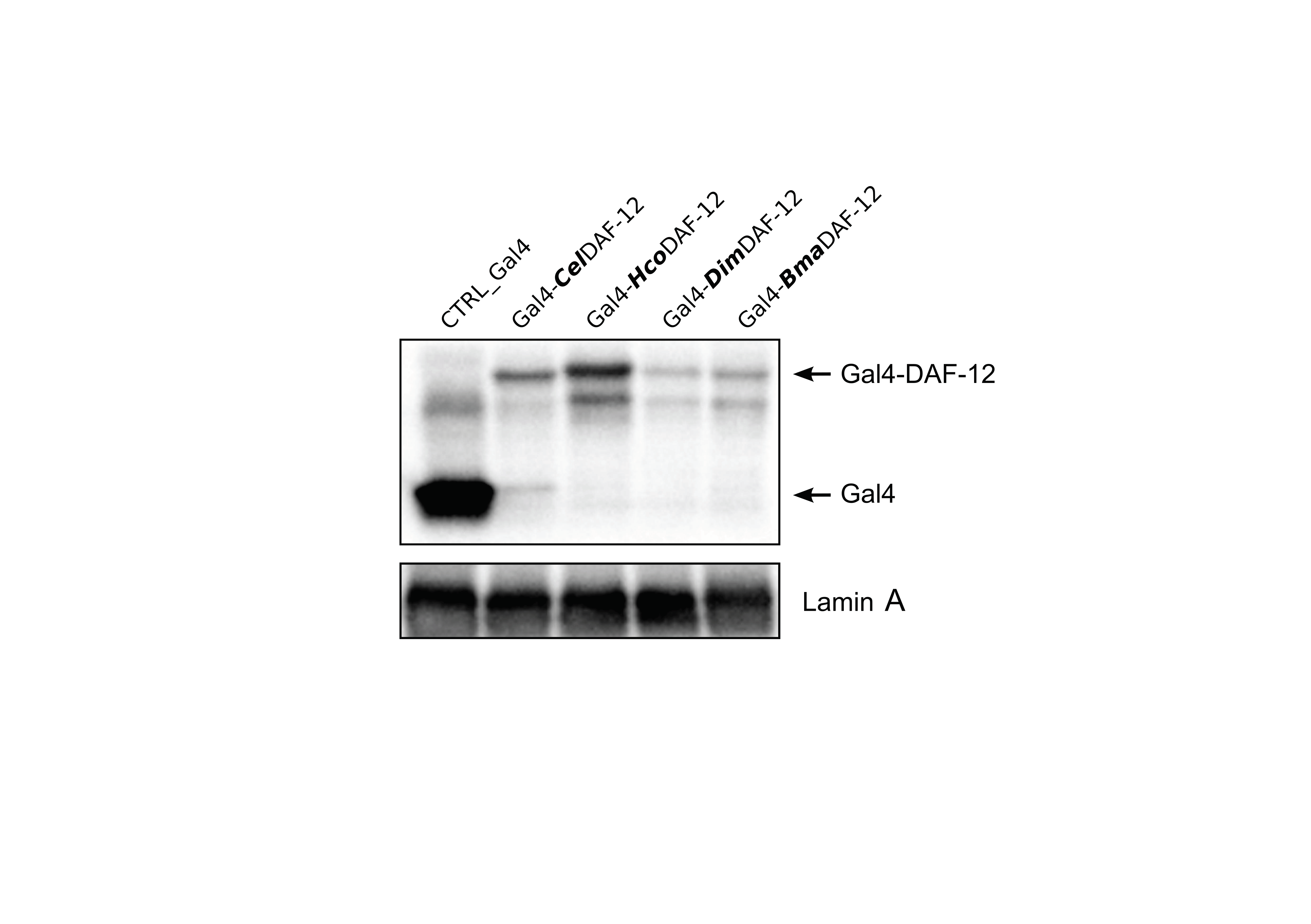

Supplement: S2 Fig — Western-blot analysis of whole cell lysate of NIH3T3 transfected with the pFN26A plasmid carrying either GAL4, GAL4-CelDAF-12, GAL4-HcoDAF-12, GAL-4-DimDAF-12 or GAL4-BmaDAF-12 using GAL4 antibody and Lamin A antibody for loading control. (TIF) [file ppat.1011462.s002.tif]

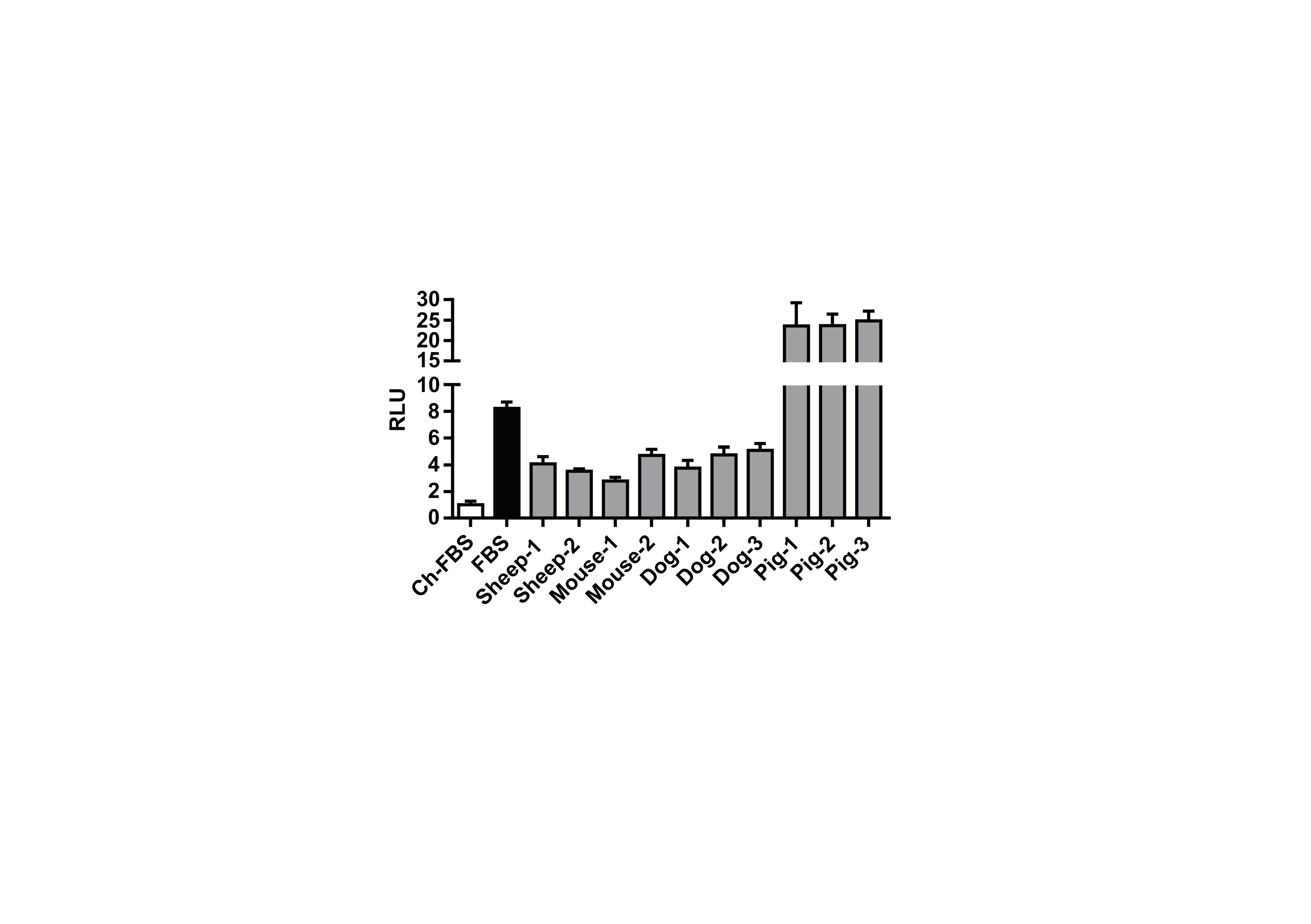

Supplement: S3 Fig — NIH3T3 cells were co-transfected with Gal4-DimDAF-12_LBD and luciferase gene reporter construct and then incubated for 24 hours with sera from different mammalian species from different individuals. Data represent the average of normalized luciferase activity and the error bars correspond to the standard deviations from three wells. (TIF) [file ppat.1011462.s003.tif]

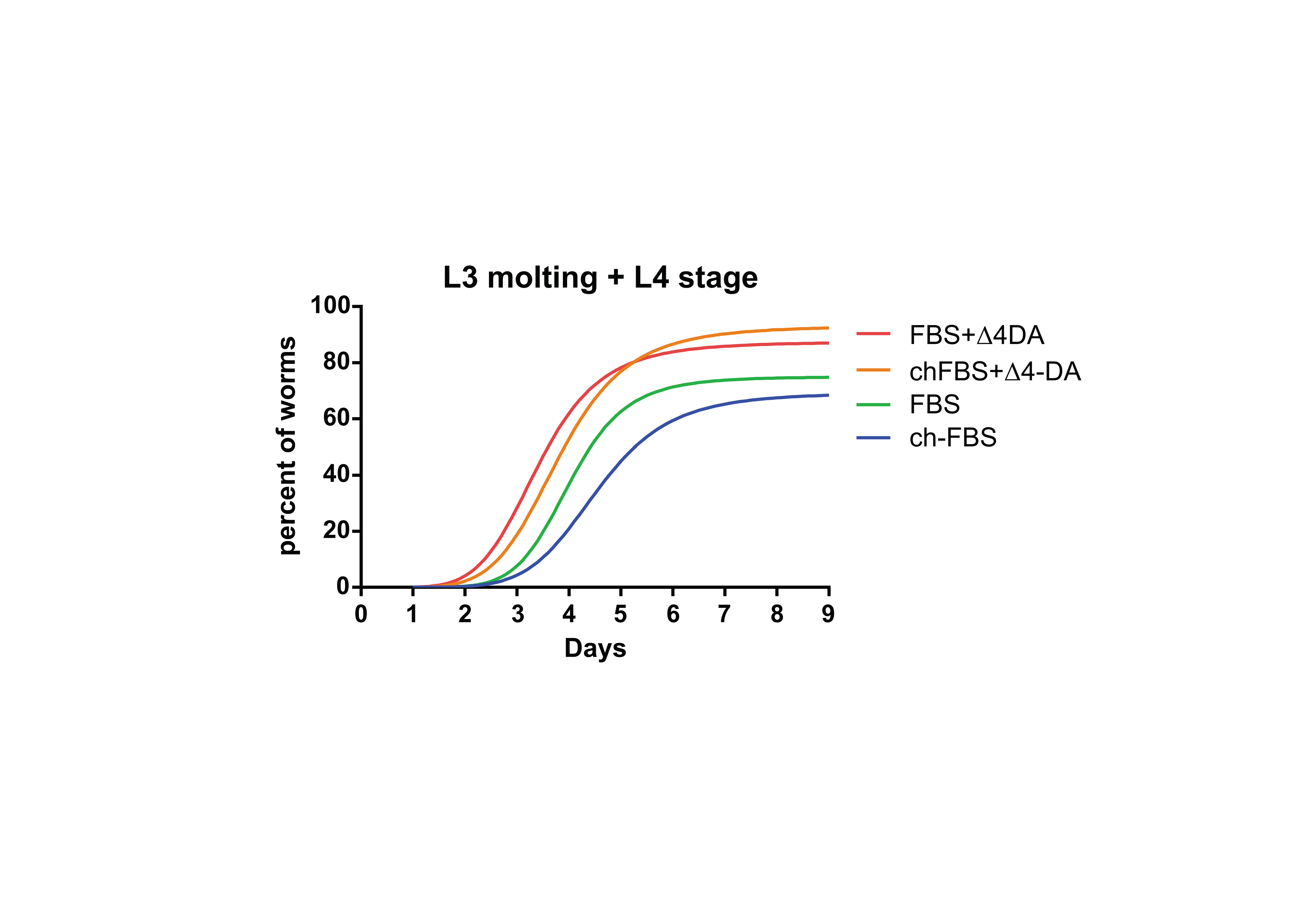

Supplement: S4 Fig — (TIF) [file ppat.1011462.s004.tif]

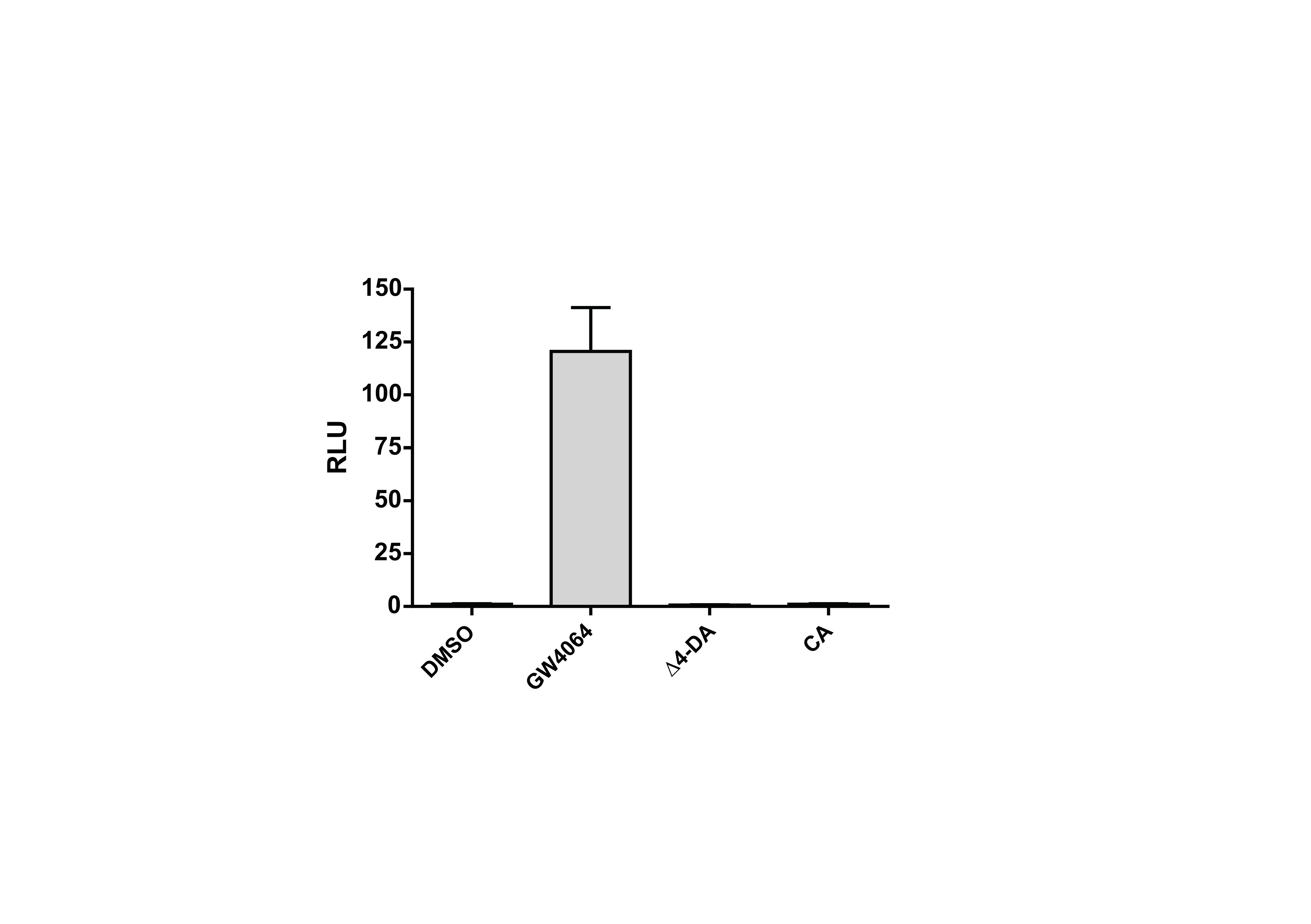

Supplement: S5 Fig — NIH3T3 cells were co-transfected with Gal4-FXR_LBD and the luciferase gene reporter construct before incubation with DMSO (0.1%) or with 10μM of Δ4-DA, CA or of the synthetic FXR agonist GW4064 for 24 hours. FXR activity was normalized to the empty vector-transfected cells and DMSO and expressed as relative light units (RLU). Data represent the average of normalized luciferase activity and the error bars correspond to the standard deviation of triplicates. (TIF) [file ppat.1011462.s005.tif]
