# Supplementary material for: Filarial DAF-12 sense the host serum to resume iL3 development during infection
Source: PLoS Pathog. 2023 Jun 20;19(6):e1011462. doi: 10.1371/journal.ppat.1011462 (PMC10313052; doi:10.1371/journal.ppat.1011462)
Supplement: S1 Table — (DOCX) [file ppat.1011462.s006.docx]

| *C. elegans* gene name | *B. malayi* gene  (% homology) |
| --- | --- |
| DAF-9 | WBGene00222818  (48.03) |
| DAF-36 | WBGene00231956  (67.60) |
| DHS-16 | WBGene00222396  (52.93) |
| HSD-1 | WBGene00227593  (44.39) |
