# Supplementary material for: Filarial DAF-12 sense the host serum to resume iL3 development during infection
Source: PLoS Pathog. 2023 Jun 20;19(6):e1011462. doi: 10.1371/journal.ppat.1011462 (PMC10313052; doi:10.1371/journal.ppat.1011462)
Supplement: S2 Table — * Tm have been calculated with the NEB Tm calculator for the Phusion Hot Start Flex DNA polymerase Buffer. For oligos containing restriction sites for cloning, Tm values for full length as well as for the sequence identical to the target are given. (DOCX) [file ppat.1011462.s007.docx]

**S2 Table**

| **Primer’s name** | **Sequence (5’-3’)** | **% GC** | **Tm*** |
| --- | --- | --- | --- |
| *Bma*DAF12Fw | ATGGCTGATATGAATAGCTTATTGTC | 26.7 | 59 |
| *Bma*DAF12Rv | TTACTAAGTAGTTTTGAAGAATTCTTTTGG | 34.6 | 59 |
| *Hco*DAF12Fw | ATGGTGGACGTTAATAATCTACTC | 37.5 | 58 |
| *Hco*DAF12Rv | CTATTCGACTTTGAAGAACTCACG | 45.2 | 57 |
| *Dim*DAF12-LBD-PvuI-Fw | CTAAGCGATCGCTAATTATCAACTTAATTCAGCCGAAC | 39.5 | 57/68 |
| *Dim*DAF12-LBD-PmeI-Rv | GCGCGTTTAAACTTAAGTAGTTTTGAAGAATTCTTTCGG | 35.9 | 57/68 |
| *Bma*DAF12-LBD-PvuI-Fw | CATACGATCGCATATCAACTTAATTCAGCTGAACTTCG | 39.5 | 59/68 |
| *Bma*DAF12-LBD-EcoRI-Rv | CTCGGAATTCCTAAGTAGTTTTGAAGAACTCTTTTGGAAG | 37.5 | 59/68 |
| *Hco*DAF12-LBD-PvuI-Fw | CTAACGATCGCCAATTATCAACTGAATGCCGC | 48.4 | 59/68 |
| *Hco*DAF12-LBD-EcoRI-Rv | GCTTTAAATCTCTGTAGGTAGTTTG | 44.1 | 59/68 |
| *Cel*DAF12-LBD-PvuI-Fw | CTAACGATCGCCAATTATCAACTGAATGCCGC | 46.9 | 58/68 |
| *Cel*DAF12-LBD-EcoRI-Rv | GCACGAATTCCCTATTTGATTTTGAAAAATTCTCCTGG | 36.8 | 58/68 |
