# Supplementary material for: Filarial DAF-12 sense the host serum to resume iL3 development during infection
Source: PLoS Pathog. 2023 Jun 20;19(6):e1011462. doi: 10.1371/journal.ppat.1011462 (PMC10313052; doi:10.1371/journal.ppat.1011462)
Supplement: Attachment — Submitted filename: Responses_to_reviewers.docx [file ppat.1011462.s008.docx]

**Response to reviewers**

**Reviewer #1**

This manuscript, entitled “Filarial DAF-12 sense Δ4-dafachronic acid in host serum to resume iL3 development during infection”, describes the characterization of DAF-12 orthologues isolated from *B. malayi* and *D. immintis* in vitro. The authors made several interesting observations, including that the filarid DAF-12 ligand binding domains (LBDs) are more sensitive to Δ4-DA than those from *C. elegans* and *H. contortus*, and present evidence that iL3 detect and are stimulated by host DAs during infection. The manuscript is interesting and makes some provocative conclusions that are not completely supported by the data. There are several minor and major issues with the manuscript that preclude a recommendation for publication. Specifically:

**Major Issues: Key Experiments Required for Acceptance**

1. Does serum from other species (e.g. pig) induce molting and development?

2. Effect of delta 4 alone on molting.

3. Effect of DA synthesis pathway inhibitors on development

We thank the reviewer for the careful reading of our manuscript and his/her wise advises. We acknowledge that our conclusion might have been a little provocative regarding the results presented originally in our manuscript. In the new manuscript, additional data have been provided that support more closely our conclusions. We have revised the conclusions to avoid any over-interpretations as suggested by the reviewer. We hope that as it is, the discussion makes the interpretation of the data relevant and clear.

1. A major conclusion is that DAs are directly sensed by *D. immitis* L3 and are determinants of activation of the larvae. However, the data clearly show that DA is a relatively minor contributor to activation and are unlikely to be the major signal that initiates development, as significant molting and development occurs in charcoal stripped serum. This is also supported by the non-specificity of serum to activate DAF12 and to promote development. Sera from multiple non-permissive hosts (including FBS) stimulate DAF-12, suggesting that the activating effect of the serum is not a host determining factor. The authors do not show the effects of sera from other species that bind and activate DAF-12. For example, pig serum activates DAF-12 in cell culture, but were not tested for its effect on molting and development. This is an important missing experiment.

We agree with the Reviewer that our data support that DA is only a partial contributor of *Dim* development. Indeed, in the *in vivo* assay performed with charcoal-stripped sera (from which DAF-12 ligands were removed), we observed substantial growth of *D. immitis* iL3 to L4 from 4 to 8 days. This is consistent with only a partial contribution of DA to this transition. This was also observed for *Hco*, for which addition of exogeneous DA only weakly stimulates its development, while inhibition of endogenous DA synthesis partially diminishes it (Ma, G et al. Plos Path 2019).

Besides this partial overall contribution of DA on *Dim* iL3 development, we also observed in our experiment, that charcoal stripped serum led to a strong significant inhibitory effect on the first period of the development, with 50% more development in FBS compared to charcoal stripped FBS at day 4 (In order to highlight this important time-period, we plotted the effect of serum and DA on iL3 development specifically at 4 days (see new Fig. 6C presented below). Moreover, larvae cultured in charcoal-stripped serum accumulate a development delay that is never caught up. Since the developmental defect in charcoal-stripped FBS is fully complemented by the addition of exogenous DA (similar development between ch-FBS+DA and FBS+DA) we propose that the significant enhancement of iL3 development that we observed on day 4 with regular FBS is mediated by DA through DAF-12 activation, the absence of which may compromise the ability of parasites to establish. **Indeed, this early development period is likely critical for the parasite’s establishment in a mammalian host.** In this context, it is important for *Bma*, that the larvae which are engaged in development (at the L3-L4 molting stage), and not the infective larvae (iL3), release excretory vesicles containing products that modulate the host immune system (Bennuru, S et al., *Plos NTD*, 2009). Consequently, by increasing the developmental rate of the infective larvae, DA from the mammalian sera may accelerate their capacity to modulate the host immune system to favor the establishment and development of the parasite.

We have added the results comments at lanes 351-352 and we have improved the discussion of this specific aspect in the revised manuscript at lanes 499-517.

**Figure 6C. Charcoal-stripped FBS significantly impairs *Dim*iL3 development after 4 days in culture.** The iL3 stage larvae were collected from mosquitos and incubated in regular or charcoal-stripped FBS containing medium in the presence or absence of Δ4-DA. The iL3 undergoing molting or which had completed the molting from iL3 to L4 were scored at day 4 under a microscope. The values plotted are the sum of the two effects: L3 molting + L4, and are the mean of 12 replicates with standard errors of the mean from 3 different pools of iL3s graphed using Prism 6.0 (Graph Pad Software, Inc.). The significance of the effects was analyzed by unpaired Student’s t-tests, *p < 0.05.

We agree with the reviewer that our data showing the particularly high effect of pig serum on DAF-12 activation is interesting and may deserve more attention. However, we emphasize that we have not argued that the mammalian host serum effect on initial development is key to host specificity. We could have hypothesized that should the pig contain higher levels of DAF-12 ligands it would make the pig a more permissive host for *D. immitis*. But it is likely that a number of factors (including parasite factors and host immunity) determine relative host specificity, and in this manuscript, it was not a goal to explore relative host specificity. Given that the *in vivo* Dim development assay depends on the very limited access to iL3 extracted from mosquitos, we would not have the opportunity to explore such large research domain that will demand too many experimental conditions and precious resource. Indeed, the starting material (the iL3-infected mosquitoes) was provided by the Filariasis Research Reagent Resource Center (FR3). Since FR3 is currently unable to provide iL3, we have been unable to complete further studies based on *Dim* iL3 development. Therefore, we focus on limited but important aspects regarding *Dim* larvae development. Comparing the effects of sera from different species on *Dim* development *in vivo* and correlating the effect with DA concentrations would be for future investigations.

Nevertheless, we partially verify this point by conducting a development assay in which we increased the proportion of serum from 10 to 50% in the medium of the assay. The figure below shows the cumulative number of molting iL3 and L4 formed at day 3, which corresponds to the most sensitive period to DA. The development rate was 4-fold higher in the presence of 50% compared with 10 % FBS, while the development was dramatically decreased when the FBS was charcoal stripped in both conditions (10% and 50%). Interestingly, the addition, in charcoal stripped FBS, of exogenous DA led to similar larval development to that obtained with 50% FBS. This is consistent with the presence of a DAF-12 ligand that activates the development of *Dim* iL3 in a dose dependent manner and which is removed by charcoal stripping. This result corroborates that the presence of DA in mammalian serum boosts *Dim* iL3 development *in vitro*. We added this figure in supplemental information.

**Increased proportion of FBS sustains *Dim* iL3 development after 3 days in culture.** The iL3 stage larvae were collected from mosquitos and incubated in 10% or 50% regular or charcoal-stripped FBS containing medium in the presence or absence of Δ4-DA. The iL3 undergoing molting or which had completed the molting from iL3 to L4 were scored at day 3 under a microscope. The values plotted are the sum of the two effects: L3 molting + L4, and are the mean of 2 replicates (for the 50% FBS conditions) or 4 replicates (for the 10% FBS conditions) and graphed using Prism 6.0 (Graph Pad Software, Inc.).

In order to address these issues in the manuscript, we modified accordingly the discussion to stress (1) charcoal treated serum did not completely abolished iL3 development (lanes 518-519) and (2) the immuno-modulatory products contained in excretory vesicles from developing larvae that likely play also an important role in the parasite establishment and for the host-specificity (lanes 509-514). As an interesting example, *Dim* larvae develop to some point in humans (whose serum contains DA) but they do not complete the full lifecycle. This may be because immunomodulatory molecules from the parasite are not fully adapted to humans.

2. Perhaps equally interesting is what other stimuli are present in stripped sera. Stripping serum is likely removes more than just sterols and DA. The potential roles of the remaining serum components are unknown, and not addressed by the authors.

Indeed, factors other than DA and sterols are removed from the sera by charcoal stripping; these include non-steroid hormones and some proteins (Cao, Z. et al., *Endocrine Research,* 2009). However, even though we cannot rule out that these factors influence *Dim* L3 molting, we showed that the effect of charcoal stripping on *Dim* development can be rescued by the addition of exogenous DA. This shows clearly that from the factors removed by charcoal stripping, DA, out of other putative DAF-12 activating ligands, is a principal factor influencing Dim iL3 development in our experimental conditions (Fig 5). Another DAF-12 activator, contained in the serum, is cholestenoic acid. But it is much less potent than DA.

3. What is the effect of Δ4-DA alone on molting? The authors referenced a previous study, but this control should be included in the current experiments.

Incubating iL3 with Δ4-DA alone would be hazardous, since most of the L3 cultured without serum died in our experimental conditions. Therefore, assessing whether DA alone would facilitate *Dim* iL3 development, in the absence of other components important for basal culture condition, leads the iL3 to death.

4. The experiments reported in figure 6 are intriguing, and the interpretation may be correct, but transcript levels do not always reflect protein levels. Does inhibition of DA synthesis prevent molting and/or development? This would confirm whether DA synthesis was required during infection or if host sterols are the initiating factor.

We agree with the reviewer that the presence (or absence) of a particular transcript not always directly correlates with the level of the corresponding protein. We keep in mind that after RNA synthesis many processes may lead to RNA degradation, or once synthesized, proteins could be inactivated or degraded. This was also appropriately stressed by rev2 (section 10). So, in the revised manuscript, we have made clear that “*the correlation between mRNA levels and protein abundance is not mandatory*”. Instead, we did exploit the RNA-seq data from which we retrieved a picture of the RNA transcript levels that allowed us to speculate on several genes belonging to the DA/DAF-12-pathway that were coincidently regulated during the development when iL3 reach the host serum. We found this information interesting since it supports a co-regulation of genes of DA synthesis and reinforces the need for exogenous DA since the transcriptional program is unfavorable to DA synthesis by the parasite. However, this still needs to be corroborated at the protein level.

5. Similarly, on Line 411 the authors pose the question of whether filarial nematodes produce DA during L3 development. Also, on line 426 they suggest that the parasite does not actively produce endogenous DA. This could be tested with DA synthesis inhibitors, i.e. do inhibitors prevent the development stimulated by serum or stripped serum?

To test the importance of endogenous DA synthesis in molting, dafadine has been used in *Cel* or *Hco* to block DA production and claimed to be a specific inhibitor of *Cel*DAF-9 and also of *Hco*DAF-9 (Luciani et al., *Nat Chem Biol*, 2011; Ma, G et al., *Plos path*, 2019). The use of such an inhibitor could be informative on the role of endogenous DA for *Dim* L3 molting, although we cannot predict if dafadine would specifically and fully inhibit *Dim*DAF-9. However, we used ketoconazole at 50 µM, which is a broad-spectrum cytochrome p450 inhibitor within the range of concentration (35-300µM) used for other parasitic nematode to block their development (Albarqi, M et al., *Plos path*, 2016; Wang, Z et al., *PNAS*, 2009; Ching-Cheng Huang, S et al., *In J Parasitol*, 2010). In our experimental conditions, we observed that all iL3 died in the presence of ketoconazole. Unfortunately, this does not allow us to conclude about the importance of endogenous DA production.

6. Line 439: One might actually conclude that other signaling pathways are involved given the relatively minor effect of DA on serum stimulated development.

We agree that factors other than exogenous DA could influence the L3 to L4 development of parasitic filarial nematodes, as previously reported for other parasitic nematodes. For example, *Dim* and *Hco* are both extremely sensitive to temperature to trigger molting (Sommeville, R et al. *The Journal of Parasitology*, 1966 ; Abraham, D et al., *Exp Parasitol*, 1990). In the case of *Hco*, increased temperature is sufficient to elicit endogenous DA synthesis which supports molting. We think that temperature could also be important for *Dim,* but that exogenous DA participates in the early activation of DAF-12 upon infection to accelerate larvae development and molting as presented in Fig 5. Nevertheless, some endogenous DA synthesis may occur, stimulated by environmental cues such as temperature. We address this point in the discussion (lanes 521 to 529).

7. Line 467: The authors need to justify how DAF-12 antagonists would prevent establishment given that worms develop without its ligand.

Indeed, in our experiment, dim iL3 larvae can develop without exogenous DA being added, in the presence of serum. The important information is that exogenous DA accelerates iL3 development, and it increased importantly the at day 4 (see new fig 6C). However, some DA may be endogenously produced which could account for the remaining (but slower at day 4) development. A DAF-12 inhibitor could abolish DAF-12 activation by exogenous or endogenous DA. Based on our data that DA accelerates the early molting, we speculate that DAF-12 inhibitors would, at least, slow down the early larvae development and impair some crucial upstream steps of infection that are likely to alter the timely development of the parasite in the host, and its final success in developing to the pathogenic adult stage. For example, slowing larvae development could impair their ability to modulate and resist the host immune system, as immune-modulatory factors may only be excreted by larvae developing optimally. Nevertheless, our interpretation is based on *in vitro* assays, and definitive conclusion would follow after experiments conducted on infected hosts, in future studies. The possibility to use DAF-12 inhibitors to prevent early establishment of parasite is justified and conclusions in the discussion of the revised manuscript have been tempered (lanes 572-578).

8. Table 2. There are insufficient details regarding how the EC50s are calculated. The authors should include the SE of the logEC50 and the number of biological and technical replicates in the table.

We apologize for forgetting details about the missing information in Table 2. We have included the number of technical replicates (3) and the SE of the logEC50 in the table 2 and its legend of the revised manuscript.

**Reviewer #2**

This manuscript posits some intriguing hypotheses on the mechanism(s) by which filarial iL3 activate inside the mammalian host; however, it seems to suffer from over-interpretation of the data presented. I would strongly recommend additional experiments (outlined below) in addition to revisions of the interpretations.

We agree with the reviewer 2 that we had over-interpreted some of our data. In the revised manuscript we have thoroughly revised the interpretations and discussion of our data, taking care not to over-interpret. We also performed additional experiments and present now new data which strengthen our hypotheses.

1. C. elegans has seven daf-12 isoforms, S. stercoralis has three daf-12 isoforms, and D. immitis has two daf-12 isoforms. Is there more than one daf-12 isoform in either B. malayi or H. contortus? Is there any conservation of daf-12 isoforms between clade III, IV, and V nematodes? Which isoform was cloned and used in this study? These are pertinent questions that should be answered.

The conservation of DAF-12 isoforms between different nematode clades is an interesting question that deserves more attention but here we have isolated and cloned only one isoform of *Bma*DAF-12 and *Hco*DAF-12. These isoforms correspond to the full-length DAF-12 in *Cel* (WormBase ID: F11A1.3a.1). Moreover, we used only the Ligand Binding Domain (LBD), and this domain is, so far as we know, conserved in all DAF-12 isoforms in *Cel* and *Dim*. We clarified this point in the materials and methods.

2. In Figure 1, why are the ligand binding domains aligned separately for B. malayi & D. immitis (panel B) and then for C. elegans & H. contortus (panel C)? Why not align all of the LBDs together in a single panel, similar to A? Also, the white amino acid letters are difficult to read on the black background.

We aligned separately the LBDs of *Bma*/*Dim* and *Cel*/*Hco* to highlight the high amino acid sequence conservation between *Bma* and *Dim*. Conversely, comparing *Cel* and *Hco* points that they are more divergent. These comparisons are completed by the pairwise amino acid sequence alignment with % homology of the 4 LBD sequences, provided in table 1.

As suggested by the reviewer 2, a comparison of the 4 LBD sequences is now presented in a single panel in S1 Fig, and interestingly this highlights the high conservation of some of the amino acids between the 4 DAF-12 species. However, when compared in pairs, the LBD of the two filarial DAF-12 appear much more conserved than those of the non-filarial DAF-12 (Fig. 1B and C).

*Bma*DAF-12 YQLNSAELRALDIVRDAFACMNEPIEDSRKAAYLKKVTHDPTDILNIIDITMRRLVKMAKKLPAFNDLSQ 705

*Dim*DAF-12 YQLNSAELRALDIVRDAFACMNEPIEDSRKASYLKKATHNPTDILNIIDITMRRLVKMAKKLPAFNDLSQ 656

*Hco*DAF-12 YQLNSAELQALDLVQEAFKGMNDPMEQGRQKMSFLKNEKSPADIMNIMDVTMRRFVKMAKKLPAFNELSQ 513

*Cel*DAF-12 YQLNAAELKALDAVREAFYGMDDPMEQGRQMQSFLKANKTPADIMNIMDVTMRRFVKVAKGVPAFREVSQ 580

*Bma*DAF-12 DGKFALLKGGMVEMLTMRGVTRFDMDRKCWRTPVVSDDSKISLE---MFDQLKEGLRDRQKEGFLKFCES 772

*Dim*DAF-12 DGKFALLKGGMVEMLTMRGVTRFDMDRKCWRTPVIPEESKISLE---MFDQLKEGLRDRQKEGFMKFCES 723

*Hco*DAF-12 DGKFALLKGSMIEMLTVRGVRRFDSSSGAWTTPTLKESCEVSIN---MFDQLNADVRSEQKMRFLQFFKI 580

*Cel*DAF-12 EGKFSLLKGGMIEMLTVRGVTRYDASTNSFKTPTIKGQ-NVSVNVDDMFAKLNANAQA-QKAKCLEFFGF 648

*Bma*DAF-12 LHPDLRNNELAIDLIVLILLFDPNRDALLDPADRITVVRHCQEYQALLHRYMESMYGHEARSRYEHLPES 842

*Dim*DAF-12 LHPDVRNNELAIDLIVLILLFDSNRDALLDPADRIIVVRHCQEYQALLHRYMESMYGHEARLRYEHLPES 793

*Hco*DAF-12 FHEDIRSNELVISMIMLIVLFSP-RDCITDPEDRRIIARHHEQYCALLNRYLESLYGDDAHKLNEQLPTA 649

*Cel*DAF-12 FDEEIKKNELAVYLVMLAVLFSVRSDPPMNENDVRIVTERHNHFMSLLNRYLESLFGEQARRIFERIPKA 718

*Bma*DAF-12 LRILRTISQNAITLFLGRVDPNQSEALPKEFFKTT 836

*Dim*DAF-12 LRILRTISQNAVTLFLGRVDPNQSEALPKEFFKTT 828

*Hco*DAF-12 LRMLREISASCGVLFLGTVNTSEAEPLPREFFKVE 684

*Cel*DAF-12 LGLLNEIARNAGMLFMGTVRSGEAEELPGEFFKIK 753

**S1 Fig. Sequence alignment of the LBD from Cel, Hco, Bma and DimDAF-12.** Multiple sequence alignment was performed with the MAFFT program from MyHits. Identical (black) and similar (grey) residues have been colored coded with the Color Align Conservation program.

We apologize for difficulties to read the white letters on the black background in Fig. 1. The uploaded figure is now optimized for reading.

3) Lines 128-129: Without an experiment to test this, I do not think the authors can claim that the DAF-12 DNA-binding domain has a “similar binding mode of the receptors on promoter binding sequences.” There are three amino acid differences in the DNA-binding domain and the impact these have on recognizing target sequences is unknown.

We agree that we do not have any concrete evidence on the binding mode of filarial DAF-12 DBD on gene promoters. The only support we have is the amino acid sequence of the DBD, which shows only small differences. But at this stage, we do not know that these differences impact DNA binding. Therefore, the sentence was changed to be less assertive, “*All DBD shared high identity (95-99%) (Fig 1A), suggesting that the proteins have closely related binding modes on the promoter regions of the target genes in the four nematode species*”.

4) Figure 2. The absolute RLU for *Hco* and *Cel* constructs is nearly twice as high as the RLU for Dim and *Bma* constructs. It is be important to show that similar levels of fusion protein are being made for these four constructs; presumably they were not codon optimized for expression in NIH/3T3 mammalian cells and thus some of the apparent differences in activity could actually be a result of different levels of protein expression. An immunoblot would be an important confirmation step. Additionally, please make it clear in the figure that these are ligand binding domains (e.g., DimDAF-12-LBD), and in the figure legend that these are a fusion proteins of the GAL4-DBD with the nematode LBD.

We thank the reviewer for having stressed that as the DAF-12 sequences of nematodes used for expression in mammalian cells were not codon optimized, this may lead to different transformation efficiency and protein expression levels. Western-blotting with an anti Gal4 antibody revealed clearly that *Cel* and *Hco*DAF-12 exhibit higher expression than *Bma* and *Dim*DAF-12. This is consistent with higher RLU observed for *Cel* and *Hco*DAF-12 than for *Bma* and *DimDAF-12* with the transactivation assay. The result of the western blot has been included in the revised manuscript as S2 Fig.

**S2 Fig. Expression of DAF-12_LBD from different nematode species fused to the DBD of GAL4 in NIH3T3 cells.** Western-blot analysis of whole cell lysate of NIH3T3 transfected with the pFN26A plasmid carrying either GAL4, GAL4-*Cel*DAF-12, GAL4-*Hco*DAF-12, GAL-4-*Dim*DAF-12 or GAL4-*Bma*DAF-12 using GAL4 antibody and Lamin A antibody for loading control.

5) Figure 3. It is really unclear how physiologically relevant the activation of *Dim*DAF-12 LBD and *Bma*DAF-12 LBD by FBS is. The assay is the same as in Figure 2, yet the RLU (which maxes out at 8) are approximately at the level of detection for the presumed endogenous dafachronic acid ligands (which max out at 80-100 RLU). These data indicate that the steroid hormones in non-stripped FBS are not native ligands for *Dim*DAF-12 or *Bma*DAF-12. These experiments need to be repeated with the additional control: charcoal-stripped FBS + cholesterol (Hoflinger et al. 2021 reports ~2 mg/ml total cholesterol in human serum). If the marginal activation of the Dim and *Bma* DAF-12 LBDs is due to non-specific binding of sterols, cholesterol would presumably active them similar to FBS; contrarily, if there is a sterol that is a relevant ligand for *Dim* and *Bma* DAF-12 LBDs, which is just at a very low concentration in FBS, then cholesterol should be similar to charcoal-stripped FBS.

We would like to point out to the reviewer that cells for the transactivation assay were cultured in the presence of 10% FBS. Assuming that the DA concentration in serum is around 4 nM (please see new Table 3), the cells were exposed to approximately 0.4 nM DA in our experimental conditions, the filarial DAF-12 ligands are ten times less concentrated than if we used 100% FBS. This is confirmed by the new experiment presented below (answer to question 12) where mouse serum activates *Dim*DAF-12 with the same strength as 0.38 nM of DA in charcoal stripped mouse serum. Moreover, we have quantified cholesterol in FBS and Ch-FBS and found similar concentrations (0.74mmol/L and 0.7mmol/L respectively).

6) Figure 4. Was the serum from various mammalian species from a single individual? A pool of individuals? Or each replicate contained serum from a different mammalian donor? I am not certain that this experiment takes into account relevant biological variation between individual mammalian organisms.

In Fig. 4, each data point corresponds to a single mammalian donor. To assess the biological variation between individuals we have performed a *Dim*DAF-12 transactivation assay with two or three donors depending on the mammalian species. There were not strong variations between individuals of the same species. These results are now presented in S3 Fig

**S3 Fig. Mammalian sera from different species and multiple donors activate DimDAF-12.** NIH3T3 cells were co-transfected with Gal4-*Dim*DAF-12_LBD and luciferase gene reporter construct and then incubated for 24 hours with sera from different mammalian species from different individuals. Data represent are the average of normalized luciferase activity and the error bars correspond to the standard deviations from three wells.

7) Lines 245-248. I am uncertain how relevant Emax is for this experiment, since the experiment was stopped after 8 days, and the percentage of larvae molting was continuing to increase between days 7 and 8 for all conditions. I recommend removing the references to Emax, unless this experiment is repeated and the number of days extended for several additional days until no further increases in activation are observed.

Even though the estimated Emax are not the most relevant parameter to discuss the effect of serum charcoal stripping on *Dim* development, it is used to calculate the T_50_ which showed significant differences when experiments were performed in the absence or presence of DA. Emax are calculated from a nonlinear mixed effects model and are therefore extrapolated from the actual data. Thus, even though larvae seem to slightly continue to develop at day 8, the data are very close to the asymptote and reliable Emax can be calculated. We have added the fitting curves calculated from the nonlinear mixed effects model as S4 Fig.

**S4 Fig. Fitting curves from a nonlinear mixed effects model of the percent of developing worms as a function of days corresponding to the data presented in Figure 6B.**

8) Line 260 and Lines 569-572: The homologs of daf-36, dhs-16, hsd-1, and daf-9 should be identified using both forward and reverse BLAST searches. I.e., the top hits of C. elegans queries should be used to search the B. malayi genome, and the predicted B. malayi proteins should be used as queries to search the C. elegans genome. The authors need to determine whether these are one-to-one homologs or merely genes encoding that particular family of proteins. Any discrepancies should be resolved with phylogenetic analyses. These experiments need to be performed and described.

We acknowledge that the homologs should have been identified both by forward and reverse BLAST search. So, we have used such an approach to verify our findings, as suggested by the reviewer. By doing this, we confirmed our previous findings for the putative homologs of daf-36, dhs-16, hsd-1, and daf-9 in *Bma*. We now mention this more appropriate approach in the materials and methods of the revised manuscript.

We acknowledge that BLAST searches are imperfect ways to identify homologs. The optimal mean to determine the homolog of a gene is to clone the putative homolog gene from parasite tissues, to express the encoded protein and to test, *in vitro*, if it shares the same enzymatic activity as the *C. elegans* protein. This has been elegantly done by Wang et al. (Wang, Z. et al., *eLife*, 2021) for *Sst daf-9*. This was a huge part of the work presented in their article. Such strategy could not be applied here. Rather, we decided to search on the *Bma* genome, the key genes of the dafachronic synthesis pathway, using sequences of *C. elegans*. Because we have retrieved simultaneously all homologue genes of interest, this encourages us to believe that the DA synthesis pathway exists in *Bma*. The fact that they all appear to be coincidently regulated at the RNA expression level reinforced that this could reflect an integrative view of the DA synthesis status. Even though imperfect, our strategy clearly draws a picture where dafachronic acid synthesis enzyme genes seems to be down regulated at the transcriptional level at the time of infection. This suggests that the global regulation of the pathway is downwards and that there is no sign of transcriptional stimulation of the main genes involved in DA synthesis.

9) Figure 6 and Line 277: Standard deviation cannot be calculated from only two replicates (minimum of three biological replicates required). Please correct this in the text and the figure; I would recommend simply plotting both data points with no error bars. Since biological variance cannot be calculated using two replicates, it is not possible to determine any statistically significant differences. Also, please provide either a reference or evidence for *B. malayi* lit-1 as a direct target of B. malayi DAF-12.

We agree with the reviewer that standard deviation cannot be calculated from only two replicates. We changed this by showing the 2 individual value points analyzed for the gene expression level as well as the mean values.

Even though there were not enough data points to provide statistics, the huge differences between the different stages appear interesting.

*Cel*Lit-1 is a well-recognized target of *Cel*DAF-12 (Shostak, Y et al., *Genes and dev*, 2004)). We retrieved a gene homolog of Cel*Lit1* in the *Bma* genome. Nevertheless, so far there is no functional evidence that *Bma*Lit-1 is a direct target of *Bma*DAF-12. We speculate, regarding the presence of homologs of several gene cascades, and the sequence homology, that *Bmal*it1 could be a target of *Bma*DAF-12.

10) Lines 279-288. The authors appear to assume that the presence or absence of a particular transcript directly correlates with the presence or absence of the protein. However, in studies of other parasitic nematodes with paired RNA-Seq and mass-spec data for the same developmental stage, there is little correlation between mRNA abundance and protein abundance. This is particularly anticorrelated for iL3, which store both proteins and mRNAs. Without any data to support or contradict DAF-9 activity in iL3, I would recommend against making any statements about whether or not iL3 produce dafachronic acids upon encountering a host.

We agree with the reviewer that the presence (or absence) of a particular transcript not always directly correlates with the level of the corresponding protein. We keep in mind that after RNA synthesis many processes may lead to RNA degradation, or once synthesized, proteins could be inactivated or degraded. This was also appropriately stressed by rev1 (section 4). So, in the revised manuscript, we have made clear that “*the correlation between mRNA levels and protein abundance is not mandatory*”. Instead, we did exploit the RNA-seq data from which we retrieved a picture of the RNA transcript levels that allowed us to speculate on several genes belonging to the DA/DAF-12-pathway that were coincidently regulated during the development when iL3 reach the host serum. We found this information interesting since it supports a co-regulation of genes of DA synthesis and reinforces the need for exogenous DA since the transcriptional program is unfavorable to DA synthesis by the parasite. However, this still needs to be corroborated at the protein level.

11) Lines 288-290 and 365-375. If filarial iL3 rely on delta4-dafachronic acid (formal name: 3-oxo-cholest-4-en-26-oic acid) for their activation, and Hoflinger et al. 2021 (ref. 17) identified 3-Oxocholest-4-en-(25R)26-oic acid at a concentration of ~3 nM in human serum, why doesn’t human serum activate either DimDAF-12 LBD or BmaDAF-12 LBD (~3 RLU in Fig. 4) even close to the extent that delta4-dafachronic acid does (at EC50 1.2 – 1.7 nM; Table 2; ~50 RLU in Fig 2)?

The DAF-12 transactivation assays were performed in the culture medium containing 10% serum. Thus, the DAF-12 ligands present in our in vitro assay are diluted ten-fold, comparing to what could be expected in the mammalian host blood, where parasites are located. This obviously decreases significantly the observed RLU measured in 10% serum-containing medium. To exemplify this, we performed experiments spiking charcoal stripped mouse serum with DA at concentrations expected in the blood (referenced in the new Table 3). The data clearly show that adding DA (R+S stereoisomers) recapitulates fully the activation of the regular mouse serum (Fig. 5B).

**Table 3. Review of the published research measuring the concentrations of Δ4-Dafachronic Acid and Cholestenoic acid in human and mouse sera.**

| **Concentrations in Human serum (nM)** | | | |  |
| --- | --- | --- | --- | --- |
| Δ4-Dafachronic Acid | | Cholestenoic Acid | |  |
| **Mean** | Variability | **Mean** | Variability | **References** |
| **4.52** | 1.39 (SD) | **223.82** | 76.96 (SD) | Crick, PJ et al., *Mol Neurobiol*, 2017 |
| **2.51** | 1.40 -3.54 (range) | **132.01** | 105.92 - 154.14 (range) | Höflinger, P et al., *J Lipid Res*, 2021 |
| **5.28** | 1.21 (SD) | **198.69** | 42.29 (SD) | Abdel-Khalik, J et al., *J Lipid Res*, 2017 |
| **4.22** | 0.017 (technical SD) | **201.20** | 7.15 (technical SD) | Crick, PJ et al., *Clinical Chemistry*, 2015 |
|  | | | |  |
| **Concentrations in mouse serum (nM)** | | | |  |
| Δ4-Dafachronic Acid | | Cholestenoic Acid | |  |
| **Mean** | Variability | **Mean** | Variability | **References** |
| **3.43** | 1.14 (SD) | **6.42** | 3.00 (SD) | Crick, PJ et al., JSBMB, 2019 |
| **4.21** | 0.18 (SD) | **8.55** | 2.93 (SD) | Griffiths, WJ et al., *BBA - MCB Lipid*s, 2019 |

**Fig 5B. Complementation of charcoal stripped mouse serum by DAF-12 ligands.** NIH3T3 cells were co-transfected with Gal4-*Dim*DAF-12_LBD and then incubated with regular or charcoal-stripped mouse serum for 24 hours. Charcoal stripped mouse serum has been supplemented or not with the indicated DAF-12 ligands at the average concentration found in mouse serum and reported in Table 3 (0.75nM for cholestenoic acid and 0.38nM for Δ4-DA). DAF-12 activity was normalized to the empty vector-transfected cells and to the charcoal stripped serum condition and is expressed as relative light units (RLU). Data represent the average of normalized luciferase activity and the error bars correspond to the standard deviation of three independent experiments. The significance of the effects was analyzed by unpaired Student’s t-tests using Prism 6.0 (Graph Pad Software, Inc.). ***p < 0.001.

12) Overall, the authors do not provide any data supporting the assertion that the activating molecule in FBS is indeed delta4-dafachronic acid. The data presented are only correlative and the conclusions speculative. Much more convincing would be: HPLC/MS data showing that delta4-dafachronic acid is indeed in FBS (and at what concentration), HPLC/MS data showing that charcoal stripping removes delta4-dafachronic acid from FBS, and that charcoal-stripped FBS + synthetic delta4-dafachronic acid (at the concentration found by HPLC/MS) activates DAF-12 with similar efficacy as FBS. This set of experiments would be much more convincing!

We agree that measuring DA in sera is an important issue, even though we consider that six high level publications released strong evidence on human and mice DA concentrations. To be able to correlate more accurately the RLU induced by the sera, the best would be to develop a method to quantify DA in the different sera used. However, such quantification of a low concentration of biliary acids and precursors requires mobilizing specific expertise (so far, only one lab, expert in oxysterol quantification, was able to detect DA in human and mouse sera). Regarding the engagement, the novelty of the data and the putative important consequences of such information, this will be part of future studies.

Novertheless, to address if DA in serum was able to activate DAF-12, we performed an experiment in which we spiked charcoal stripped mouse serum with DA and cholestenoic acid at the concentration expected in sera (measured in mice by HPLC/MS (Crick, PJ et al.; *JSBMB*, 2019; Griffiths, WJ et al.; *BBA – MCB* *Lipids*, 2019)). Because DA possess two stereoisomers ((25R) and (25S)) that exhibit differential efficiency with *Cel*DAF-12 (Sharma, KK et al.; *Mol Endocrinol*, 2009) and because we don’t know which one is present in mammalian sera, we used (25R)-Δ4-DA alone or a racemic solution ((25R) + (25S)) (the (25S) alone is not commercially available). We show that the addition of DA at 0.38 nM (corresponding to 1/10 of serum concentration to mimic the 10% serum culture medium) partially complement charcoal stripped mouse serum activity in its (25R) conformation. Most importantly, the Δ4-DA racemic solution reestablished completely the activity of the serum while cholestenoic acid did not activate *Dim*DAF-12 at the concentration reported in the literature for mouse serum. We now present these additional data (see new Fig. 5) presented in a new result section with the corresponding discussion which suggest that DA is a good, and may be the sole, candidate for filarial DAF-12 activation by mammalian serum.

**Fig 5B. Supplementation of charcoal stripped mouse serum by DAF-12 ligands.** NIH3T3 cells were co-transfected with Gal4-*Dim*DAF-12_LBD and then incubated with regular or charcoal-stripped mouse serum for 24 hours. Charcoal stripped mouse serum has been supplemented or not with the indicated DAF-12 ligands at the average concentration found in mouse serum and reported in Table 3 (0.75nM for cholestenoic acid and 0.38nM for Δ4-DA). DAF-12 activity was normalized to the empty vector-transfected cells and to the charcoal stripped serum condition and is expressed as relative light units (RLU). Data represent the average of normalized luciferase activity and the error bars correspond to the standard deviation of three independent experiments. The significance of the effects was analyzed by unpaired Student’s t-tests using Prism 6.0 (Graph Pad Software, Inc.). ***p < 0.001.

**Reviewer #3**

This manuscript characterizes the nuclear receptor DAF-12 in filarial nematodes and claims that host serum contains a dafachronic acid (Δ4-DA) that directly activates the parasite DAF-12, which in turn promotes the parasite’s L3 quiescence exit. The authors show that DAF-12s isolated from *B. malayi* and *D. immitis*, two prevalent filarial nematodes, are activated by dafachronic acids, which is not a particularly novel or surprising discovery since these compounds have been previously shown to bind and activate DAF-12 from several other free-living and parasitic nematodes. The apparent novelty of the paper is demonstrating that sera from multiple mammals can activate the bm- and di-DAF-12s and promote filarial nematode development. Since an isomer of Δ4-DA has previously been reported to be present in human serum, the authors concluded that Δ4-DA is the physiological signal in serum that promotes filarial nematodes to exit L3 quiescence for reproductive development. The authors also profiled the mRNA expression of the homologs of known DA biosynthetic enzymes to conclude that worms do not make DA. Unfortunately, neither of these conclusions are well supported by the data in its present form.

We understand the reviewer concern about over-interpretation of our data. We thank the reviewer for pointing out the statements requiring clarification. Accordingly, to the reviewer comments we have tempered our conclusion and completed several requested experiments. We paid particular attention to interpret and discuss the results without extrapolation. We are pleased to re-submit a revised manuscript which has been straightened by direct evidences supporting that Δ4-DA is one of the physiological signal in serum that promotes filarial nematodes to exit L3 quiescence for reproductive development.

1. Evidence to support the conclusion that the host serum factor that activates filarial DAF-12 species is Δ4-DA is not directly supported by the data in this paper. Indeed, the authors present no evidence that serum contains Δ4-DA at concentrations that would activate the receptor and affect worm development. Instead, the authors cite the key previous paper (Höflinger, J Lipid Research, 2021) as evidence that serum contains Δ4-DA. However, a careful reading of that paper reveals that this Δ4-DA compound, also known as 3-oxocholest-4-en-(25R)26-oic acid, is actually the 25(R) enantiomer of Δ4-DA. In that paper, the reported concentration was 1nM. A key missing experiment is for the authors to show in their samples that the active form of Δ4-DA is present, which they did not do. Even if this compound is in the serum sample that the authors used, there are two things that still do not support their conclusion. First, the pharmacological properties of dafachronic acid ligands for DAF-12 receptors depend on the stereochemistry at the C-25 position, i.e., the (S) versus (R) form. The 25(S)-DA is at least an order of magnitude more potent than the 25(R)-DA in activating all other tested DAF-12 receptors. As noted above in the cited reference, only the 25(R) enantiomer was found, which makes sense because unlike the DAF-9 enzyme in nematodes, the 26-hydroxylase in mammals that catalyzes the 26-oxdidation favors 25(R) products (Javitt, J. Lipid Res. 1990). A direct comparison of the activities of the two Δ4-DA enantiomers is needed to make any conclusions on that point. More importantly, to demonstrate what the activity is in the serum used in these studies, the authors should chemically isolate and identify the compound in their own serum samples, which might be quite different than those reported in the cited study. The results of that experiment would be of great interest and directly address the main conclusion of the paper.

We agree with the reviewer that the activation of filarial DAF-12 by DA naturally present in the mammalian serum is still a hypothesis. DA in serum is a new information. Before our work, nobody had made the link between 3 oxocholest-4-en-26-oic acid and DA at concentrations that we have shown to activate filarial DAF-12.

This hypothesis is strongly supported by several aspects listed below and some are presented in a synthetic table. Moreover, our data were reinforced by new supportive experiments. New tables and figures are presented now in the revised manuscript.

- There are several papers that report the presence 3-oxocholest-4-en-26-oic acid which is identical with Δ4-DA in human and mouse sera at concentrations well above the EC50 of Δ4-DA for *Dim* and *Bma*DAF-12.
- The 10% serum in the cell medium, which is known to contain DA, was sufficient to activate the filarial DAF-12.
- The serum mediated activation is removed by charcoal stripping, supporting that the DAF-12 activation is mediated by a bile acid like molecule, such as DA.
- Effect of charcoal stripped mouse serum on DimDAF12 is fully rescued by DA at a concentration reported in mouse serum.

In order to make these aspects clear, we provide a new table 3 that gathers all the observed concentration of DA and cholestenoic acid from the literature. In order to make easier the comparison with our other data, concentrations are expressed in nM and not in ng/ml as in the original articles.

**Table 3. Review of the published research measuring the concentrations of Δ4-Dafachronic Acid and Cholestenoic acid in human and mouse sera.**

| **Concentrations in Human serum (nM)** | | | |  |
| --- | --- | --- | --- | --- |
| Δ4-Dafachronic Acid | | Cholestenoic Acid | |  |
| **Mean** | Variability | **Mean** | Variability | **References** |
| **4.52** | 1.39 (SD) | **223.82** | 76.96 (SD) | Crick, PJ et al., *Mol Neurobiol*, 2017 |
| **2.51** | 1.40 -3.54 (range) | **132.01** | 105.92 - 154.14 (range) | Höflinger, P et al., *J Lipid Res*, 2021 |
| **5.28** | 1.21 (SD) | **198.69** | 42.29 (SD) | Abdel-Khalik, J et al., *J Lipid Res*, 2017 |
| **4.22** | 0.017 (technical SD) | **201.20** | 7.15 (technical SD) | Crick, PJ et al., *Clinical Chemistry*, 2015 |
|  | | | |  |
| **Concentrations in mouse serum (nM)** | | | |  |
| Δ4-Dafachronic Acid | | Cholestenoic Acid | |  |
| **Mean** | Variability | **Mean** | Variability | **References** |
| **3.43** | 1.14 (SD) | **6.42** | 3.00 (SD) | Crick, PJ et al., JSBMB, 2019 |
| **4.21** | 0.18 (SD) | **8.55** | 2.93 (SD) | Griffiths, WJ et al., *BBA - MCB Lipid*s, 2019 |

We agree with reviewer 3 that the mammalian 26-hydroxylase that catalyzes the 26-oxdidation favors 25(R) enantiomer products. Thus, it is likely that at least part of the Δ4-DA found in mammalian sera is of 25(R) stereochemistry. To measure the potency of the 25(R) Δ4-DA for filarial DAF-12 we carried out a new dose response experiments with *Dim* and *Bma*DAF-12 (Fig 5A). We found the EC50 for *Dim* and *Bma*DAF-12 around 5nM for 25(R) Δ4-DA DA which is 5 times higher than that for the 25(S)Δ4-DA enantiomer. Even if 25(R) Δ4-DA is produced by mammals, it is possible that this enantiomer could be transform into the 25(S) enantiomer as 25S-epimers of other bile acid precursors have been identified in WT and CYP27A1 -/- mice (Griffiths, WJ et al.; BBA - MCB Lipids, 2019). Importantly, Pr. W. Griffith and Y. Wang did not use both 25(S) and 25(R) standards in their HPLC/MS experiments. Thus, they could not discriminate the two enantiomers. As a consequence, which one of the 25(S) or the 25(R) Δ4-DA is present in human and mouse sera is unknown.

We agree that measuring DA in sera is an important issue to validate our conclusions. However, such quantification of a low concentration of biliary acids and precursors requires mobilizing specific expertise and investments. Regarding the engagement, the novelty of the data and the putative important consequences of such information, this will separately be addressed. However, to test if Δ4-DA is the filarial DAF-12 ligand present in the mammalian sera and removed by charcoal stripping, we have performed complementation experiments of charcoal stripped mouse serum (Fig. 5B). In these experiments, we compared the activity of *Dim*DAF-12 in the presence of mouse serum or charcoal stripped mouse serum with the addition of the expected concentration of Δ4-DA or cholestenoic acid, based on concentrations reported in the literature (see Table 3 above).

**Fig 5. Consequence of Δ4-DA stereochemistry on filarial DAF-12 activation. (A)** Dose-response curves of (25R)-Δ4-DA on the activation of *Bma* and *Dim*DAF-12. NIH3T3 cells were co-transfected with Gal4-DAF-12_LBD from *Bma* or *Dim* and the luciferase gene reporter construct. Recombinant cells were incubated with increasing concentrations of (25R)-Δ4-DA. Data representing normalized luciferase activities are plotted with nonlinear regression fit using sigmoidal dose-response with variable slope (Prism 6.0, Graph Pad Software, Inc.). The Fig shows one representative experiment out of two. Values are means of technical triplicates ± standard deviation. **(B)** Complementation of charcoal stripped mouse serum by DAF-12 ligands. NIH3T3 cells were co-transfected with Gal4-*Dim*DAF-12_LBD and then incubated with regular or charcoal-stripped mouse serum for 24 hours. Charcoal stripped mouse serum has been supplemented or not with the indicated DAF-12 ligands at 10% of the average concentration found in mouse serum and reported in Table 3 (0.75nM for cholestenoic acid and 0.38nM for Δ4-DA). DAF-12 activity was normalized to the empty vector-transfected cells and to the charcoal stripped serum condition and is expressed as relative light units (RLU). Data represent the average of normalized luciferase activity and the error bars correspond to the standard deviation of three independent experiments. The significance of the effects was analyzed by unpaired Student’s t-tests using Prism 6.0 (Graph Pad Software, Inc.). ***p < 0.001.

We used the 25(R) enantiomer and a racemic solution of Δ4-DA (because no pure 25(S) stereoisomer is currently commercially available). These data support our hypothesis that DA is likely the main filarial DAF-12 ligand of mammalian sera since only Δ4-DA (25(R) enantiomer alone or the racemic solution of Δ4-DA) partially or fully recapitulates *Dim*DAF-12 activation by mouse serum.

2. The authors show that mammalian sera can activate the D. immitis DAF-12 (Fig 4) and promote L3 quiescence exit (Fig 5). They use these experiments as further evidence to support the notion that the serum activity is Δ4-DA. However, again there is no direct evidence to support this. Importantly, while charcoal stripping essentially eliminated all DAF-12 activation, it only marginally impaired L3 quiescence exit (Fig. 5), which should not have been the case if this was due to the lack of Δ4-DA. It also is notable that whatever this activity is in the transfection assay, it is much weaker than Δ4-DA. In thinking about this more, a good candidate for this activity might be cholestenoic acid or one of the other congeners that are found at much higher concentrations in serum as reported in the Höflinger, J Lipid Research paper. Cholestenoic acid is at a concentration in serum that is in the range of activity that the authors found in Fig. 2. Given that cholestenoic acid is also a poor activator of C. elegans DAF-12, this would also explain why serum did not activate C. elegans DAF-12, while it showed some activity on the filarial DAF-12s. Furthermore, since adding exogenous Δ4-DA to FBS can accelerate the L3 quiescence exit (1 day earlier, Fig 5B), it is equally if not more possible that the serum stimulates DA synthesis in the parasite, which takes some time. Taken together, all these findings further support the alternative conclusion that something in serum other than Δ4-DA may be directly, but weakly activating DAF-12, and/or that serum is also stimulating the true endogenous ligand’s synthesis. Again, without isolating the compound and showing its identity, it is not possible to draw any conclusions.

We agree that removal of DAF-12 ligands from FBS by charcoal stripping don’t completely abolish *D.* *immitis* larval development. We discussed this observation in the discussion making the point that other factors, such as temperature, may contribute to the stimulation of *Dim* iL3 development *in* *vitro* at lanes 518-522 (“*However, we noticed that incubating Dim iL3 in medium containing charcoal treated serum did not completely abolished their development. This might be due either to incomplete removal of the DAF-12 ligands from serum or to additional cues in the culture conditions which could stimulate a slower rate of filarial larvae development. One of them may be the temperature of 37 °C in the assays to mimic the host's environmental conditions since Dim iL3 do not molt at 27°C*”). Interestingly, this has also been observed for other parasitic nematodes such as *Haemonchus* *contortus*. Indeed, *H. contortus* L3 won’t develop below 38°C and addition of exogenous dafachronic acid also impacts its *in* *vitro* development (Ma, M et al.; *Plos Path*., 2019; Figure 2D) to the same magnitude as *D.* *immitis*.

Clearly, DAF-12 activation by DA is not the only determinant of parasitic nematode larval development. However, we acknowledge that the dynamic development of *Dim* iL3 which start to molt around 4 days in our experimental culture conditions, and the expression of the putative dafachronic acid synthesis pathway genes which also increases after four days, suggest that endogenous production of DA may take over exogenous DA from the serum to sustain more steadily larval development beyond the initial couple of days in the mammalian host. We rephrased this interpretation of the data in the discussion (lanes 548-552). Despite the partial overall contribution of DA on *Dim* iL3 development, we stress that in our experiment, charcoal stripped serum led to a strong significant inhibitory effect on the first period of the development, with 50% more development in FBS compared to charcoal stripped FBS at day 4 (see below the new Fig. 6C that highlights this aspect). Since the developmental defect in charcoal stripped FBS is fully rescued by the addition of exogenous DA (similar development between ch-FBS+DA and FBS+DA) we propose that the significant enhancement of iL3 development that we observed on day 4 with regular serum is likely to be mediated by DA though DAF-12 activation, the absence of which may compromise the ability of parasites to promote infection establishment. Indeed, this early development period should correspond to the installation of the parasite in the new host. Importantly, for *Bma*, the larvae which are engaged in development (L3-L4 molting stage) and not the infective larvae, release excretory vesicles containing products that modulate the host immune system to favor the parasite (Bennuru, S et al., *Plos NTD*, 2009). Consequently, by increasing the developmental rate of the infective larvae, DA from the mammalian sera may accelerate their capacity to modulate the host immune system and enhance development and establishment of the parasite. Moreover, if we agree that the activating effect of the serum is not a host determining factor (we did not propose that it was), we would like to stress that the immune-modulatory products contained in the excretory vesicles from developing larvae may play an important role in host specificity.

**Figure 6C. Charcoal stripping of FBS significantly impairs *Dim* iL3 development after 4 days in culture.** The iL3 stage larvae were collected from mosquitos and incubated in regular or charcoal-stripped FBS containing medium in the presence or absence of Δ4-DA. The iL3 undergoing molting or which had completed the molting from iL3 to L4 were scored at day 4 under a microscope. The values plotted are the sum of the two effects: L3 molting + L4, and are the mean of 12 replicates with standard errors of the mean from 3 different pools of iL3s graphed using Prism 6.0 (Graph Pad Software, Inc.). The significance of the effects was analyzed by unpaired Student’s t-tests, *p < 0.05.

As argued above for question 1, we now have evidence (Fig. 5B) that Δ4-DA (25(R) and/or 25(S)) are the main DAF-12 mammalian serum ligands at least for mouse serum. These new data further support our conclusion that Δ4-DA naturally present in mammalian serum stimulate *Dim* iL3 development, at least during the early days of infection. Moreover, as cholestenoic acid did not activate *Dim*DAF-12 at concentrations reported in the literature, in mouse serum and because it activates DimDAF-12 with lower efficiency than DA (Fig. 2 and Table 2) we conclude that cholestenoic acid may only marginally affect filarial DAF-12 activity induced by the mammalian sera.

3. A third piece of evidence presented to support the authors’ conclusion that the parasites do not synthesize DA is that the mRNA expression of a protein the authors annotate as “BmaDAF-9” is very low during the L3 quiescence exit. This however does not prove DA synthesis in the parasites does not exist. There are a couple of problems with this interpretation. First, the authors provided no conclusive evidence to show whether the “BmaDAF-9” claimed in this study is the DAF-9 ortholog. The authors make this claim based solely on bioinformatic analysis without any experimental evidence. Indeed, the protein sequence identity compared to C. elegans DAF-9 is only ~35% (by both Blastp of NCBI and Clustwal Omega of EMBL-EBI), which is too low to predict what it might do. In fact, the very logic of this strategy seems contradictory: if the authors are claiming “BmaDAF-9” is the daf-9 gene (as identified by homology) then it should function as DAF-9. As recently demonstrated in Strongyloides stercoralis, one cannot conclude the identity of the DAF-9 ortholog without testing its activity in a biochemical assay (Wang, eLife 2021). The real experiment that needs to be done is to test all of the potential cytochrome P450s in the parasite for their ability to make DA (using as substrates either 4-cholestene-3-one or lathosterone). Lastly, mRNA levels do not necessarily correlate with protein levels. This is especially true for DAF-9 where a feedback regulation of its mRNA levels by DAF-12 activation has been reported in C. elegans (Wang, Plos Genetics, 2015). A simple way to test whether these parasites synthesize DA might be to feed the parasites isotope-labeled cholesterol and then assay the incorporation of the isotope into DA has been done recently in Strongyloides stercoralis (Wang, eLife, 2021).

We acknowledge that BLAST searches are imperfect ways to identify homologs and that the most appropriate means to confidently determine the homologue of a gene is to clone it and verify that the encoded protein possess the same activity and function as the protein in *C. elegans*. This was a huge part of research that has been elegantly done by Wang et al. ((Wang, Z. et al., *eLife*, 2021)) for *Sst daf-9*. Searching for the role of enzymes in nematode parasites requires mobilizing specific expertise and regarding the novelty of the data and the importance of such information, this will be part of future research. Although we are aware that reasoning about the comparative RNA expression of potential homologous genes in the dafachronic synthetic pathway is limited in terms of information, we believe that the use of the open RNA seq database has allowed us to shed light on certain aspects related to our hypothesis. Such strategy has drawn a clear picture where dafachronic synthesis enzyme genes are concurrently down regulated at the strategic period of infection. Because such gene regulation time-profile does not concern only one isolated gene, but concerns concomitantly several genes encoding for enzymes that are functionally related, we propose that this is in favor of a low production of DA by the parasite at that specific period of infection.

We cannot exclude that the larvae produce endogenous DA. In contrast to *C. elegans*, *H. contortus* and *S. stercoralis* where hundreds of thousand nematodes were required to show endogenous DA production, it would be very challenging to get enough filarial iL3 to assess DA production at the iL3 stage.

We took into account reviewer 3 comments and have rephrased our conclusions. We discuss that there is little to no expression of putative DA synthesis genes at the time of infection (Lanes 541-546), which legitimately let us pose the question if the larvae are producing endogenous dafachronic acids upon penetrating the host. This point is consistent with the experimental evidence that exogenous DA produced by the mammalian host appears to be promoting the development of the larvae during the first days of infection.
