# Supplementary material for: Filarial DAF-12 sense the host serum to resume iL3 development during infection
Source: PLoS Pathog. 2023 Jun 20;19(6):e1011462. doi: 10.1371/journal.ppat.1011462 (PMC10313052; doi:10.1371/journal.ppat.1011462)
Supplement: Attachment — Submitted filename: Betous et al Response to 2nd revision.docx [file ppat.1011462.s009.docx]

**Responses to 2^nd^ revision, reviewer 3**

We thank the reviewer #3 for the time he/she took to raise important concerns, and to give us the opportunity to improve the message of the paper.

Please find below a detailed list of our responses to the reviewer comments, and a description of the changes we have made in the manuscript.

Reviewer #3: This revised manuscript is improved, but still does not satisfactorily justify a couple of the key conclusions of the paper. The claim in the title that “filarial DAF-12 sense Δ4-dafachronic acid in host serum to resume iL3 development” is overstated and based on circumstantial, and in some cases, contradictory evidence. While it is possible that this statement is true or partially true, this definitive statement was not proven and is based on circumstantial evidence. In addition, the authors ruling out endogenous DA synthesis as not being required is also not justified. The current data and conclusions fail to take into account other possibilities that were not ruled out as explained below:

In order to temperate the claim which was overstated, the title has been revised:

Previous title: Filarial DAF-12 sense Δ4-dafachronic acid in host serum to resume iL3 development during infection

**Revised titles**: *Filarial DAF-12 sense the host serum to resume iL3 development during infection*

Moreover, we have modified both the abstract and the conclusion of the revised manuscript accordingly to the points raised by the reviewer

1) A compound other than Δ4-DA may be present that activates development. Although the authors provide published data from others that some form of DA may be present, the fact that serum stripping eliminates all DAF-12 activity but still induces development continues to be convincing evidence for the existence of another mechanism. The assay the authors used to monitor DAF-12 activity is robust and highly sensitive for detecting even low levels of DA, so the fact that charcoal stripping removes all activity strongly suggests there is no significant ligand left in the serum. Nevertheless, stripped serum still significantly induced resumption of development. Thus, there must be something else being sensed and this may be stimulating endogenous production of DA

We agree with the reviewer 3 that regarding the current data shown in the manuscript, we cannot exclude the contribution of other environment components, that may activate iL3 development. We now mention this point clearly in the discussion:

Lines 517-521: “*However, we cannot exclude that other mechanisms are also responsible for stimulating development. Indeed, the fact that charcoal stripping of the serum eliminates all DAF-12 activity in the transactivation assay, but did not completely abolish iL3 development, suggests the existence of other environmental factors which could promote larval development in vivo, possibly through stimulating endogenous filarial DA production.”*

2) Endogenous DA synthesis may still be required for resumption of iL3 development (as has clearly been shown in other species). The authors try and argue around this by claiming that DA biosynthetic enzyme mRNA levels are low at the point of infection. No evidence was presented that any of the mRNAs surveyed actually encode a biosynthetic enzyme (or that the protein levels matched the RNA levels). However, giving the authors the benefit of the doubt that these homologous sequences do encode the responsible enzymes, their interpretation of their own data still contradicts their claim. The enzyme *Bma*DAF-9, which they claim is the DAF-9 ortholog, is very highly expressed at L3i! This is exactly what one might expect in order to jumpstart the immediate synthesis of DA upon infection. Note that the other enzymes in the upstream synthetic pathway are not rate-limiting and thus are not necessarily even needed at this point, as long as the substrate for DAF-9, which is rate-limiting, is present in the parasite (or may be acquired from the host upon infection). It is also noteworthy that as the parasite starts its developmental program, all of these enzymes increased. This also makes sense, since as has been shown in *S. stercoralis*, DA levels become highest later in parasitic development within the host. Lastly, as the authors also acknowledged, the mRNA levels do not necessarily correspond to protein levels, and this has been observed in other species also.

Regarding the parallel between RNAseq data and the L3 development, we consider the first few days of infection for our reasoning, to only discuss putative dafachronic acid synthesis enzyme gene homologs expression while the parasite is in the host.

Nevertheless, we agree with the reviewer that the RNAseq data cannot let us conclude that the parasites do not produce DA, mainly if we consider the high expression of *Bmadaf-9* in iL3 before entering the host. However, to integrate the reviewer 3 comments, we have tempered the statements on the endogenous DA synthesis during the development of L3, and modified the paragraph as follow:

Lines 541-554 : “*The low expression of the DA synthesis genes during the first days of the mammalian infection period raises the question of whether the filarial L3 larvae produce sufficient endogenous dafachronic acids to activate DAF-12. If the production of DA is low in L3 upon initial infection, DAF-12 activation would then depend mostly on the mammalian host Δ4-DA to sustain filarial parasites development. The expression of putative B. malayi homologues of daf-36, hsd-1 and dhs-16 peaked at 4 days post infection, and daf-9 between 4 and 8 days, suggesting stimulation of endogenous production of DA a few days after infection. Accordingly, the first larvae molting occurred at day 4 in our Dim development assays. During dauer exit in C. elegans, a DAF-12 dependent positive feedback loop facilitates development by ensuring that sufficient DA is dispersed throughout the body and serves as a robust fate-locking mechanism [35]. Similarly, filarial DAF-12 activation by the host Δ4-DA may feed such a feedback loop to stimulate endogenous DA production and ensure robust molting and development in the days following infection. This is consistent with observations made in S. stercoralis where DA levels become highest later in parasitic development within the host [16].”*

3) As mentioned in point #1, something in the serum may be stimulating DA synthesis. The authors suggest temperature or some other environmental factor is responsible for stimulating development, which may work without DA. This is a misinterpretation of how these environmental factors are already known to work in other species. Studies in both C*. elegans* and *S. stercoralis* have shown that these factors act upstream in the resumption of dauer/parasitic development by turning on DA synthesis (which is still required). In these and other nematode species, DAF-12 and DAF-9 are required for resumption of development, which is also convincing evidence that the ligand is required.

In order to argue on the stimulating effect of temperature on L3 development through DA endogenous production, we have changed the discussion following the new paragraph discussing the reviewer point #1:

Lines 521-525: “*Consistently, elevated temperature mimicking the host body temperature stimulates endogenous DA synthesis and iL3 developmental resumption in H. contortus [15] and S. stercoralis [16]. While DimiL3 only molt at 37°C [32], this poses the question of whether filarial nematodes produce endogenous DAs during L3 development.”*

A couple of other points are worth mentioning that the authors use to argue against endogenous ligand synthesis. First, the lack of an effect of dafadine as an inhibitor of endogenous DA synthesis in the parasite is not at all conclusive. No evidence was presented that dafadine inhibits DA synthesis in the parasite. C. elegans DAF-9 is not the same enzyme as *Bma*DAF-9 or DAF-9 found in other parasites and may not be effective against parasites. To that point, dafadine does not inhibit DAF-9 or endogenous DA synthesis in *S. stercoralis*, demonstrating the lack of broad inhibitory activity.

We agree with the reviewer that there is no evidence that the *Cel*DAF-9 inhibitor dafadine inhibits DA synthesis in filarial nematodes.

Despite these concerns, the paper is of interest if the conclusions and discussion were modified accordingly. A simple suggestion for how the authors might improve the paper to be acceptable (without further experimentation), is to fairly discuss the points above and change their definitive conclusions in the abstract and title. Why not just state “Filarial DAF-12 senses host serum to resume iL3 development” and mention the alternatives that are just as likely, or perhaps work in concert? These changes would still get their points across and would not limit their speculation pending further definitive results.

We thank the reviewer for acknowledging the interest of the manuscript. As stated above, we took into account the reviewer’s comments and we have modified the title, the abstract, the discussion and the conclusion of the revised manuscript accordingly.
